# Intention to emigrate after graduation among medical students from two universities in Lima, Peru

**DOI:** 10.1371/journal.pone.0342477

**Published:** 2026-02-20

**Authors:** Sebastian A. Medina-Ramirez, Alvaro Taype-Rondan

**Affiliations:** 1 Center for Research in Primary Health Care (CINAPS), Universidad Peruana Cayetano Heredia, Lima, Peru; 2 Grupo de investigacion P53, Facultad de Medicina Humana, Universidad Peruana Union, Lima, Peru; 3 Unidad de investigacion, Instituto Nacional de Ciencias Neurologicas, Lima, Perú; 4 Unidad de Investigación para la Generación y Síntesis de Evidencias en Salud, Vicerrectorado de Investigación, Universidad San Ignacio de Loyola, Lima, Perú; 5 EviSalud - Evidencias en Salud, Lima, Perú; Flinders University, AUSTRALIA

## Abstract

**Objective:**

To determine the prevalence of the intention to emigrate among third- to seventh-year medical students from two private universities in Lima, Peru.

**Materials and methods:**

Cross-sectional study in third- to seventh-year medical students. To assess associated factors, we calculated adjusted prevalence ratios (aPR) using Poisson regression models.

**Results:**

Among 508 medical students, 92.1% reported an intention to emigrate. A rising trend was observed within one of the universities evaluated. When analyzing factors associated with the intention not to emigrate, a lower frequency was found among students aged 23–25 compared to those aged 18–22 (aPR: 0.35; 95% CI: 0.13–0.91). Additionally, the intention not to emigrate was more common among those who had completed an external rotation in primary care (aPR: 2.10; 95% CI: 1.18–4.10), and less common among those who preferred a surgical specialty after graduation (aPR: 0.40; 95% CI: 0.20–0.82).

**Conclusions:**

Our study reveals a high prevalence of the intention to emigrate among third- to seventh-year medical students. It is essential to consider implementing policies that improve training conditions and labor opportunities in order to retain medical talent and strengthen the national health system.

## Introduction

The emigration of healthcare professionals is a growing trend that poses significant challenges, particularly for developing countries, where emigration rates are considerably higher than in high-income nations. This inclination to emigrate is often driven by factors such as limited job opportunities, restricted avenues for professional development, and the attractive economic and academic incentives offered by developed countries [1,2].

In this context, physician emigration has become a critical issue, as it results in the loss of trained personnel [3]. This weakens health systems in source countries, exacerbates inequalities in access to healthcare services, and intensifies the shortage of professionals, particularly in rural areas [4].

A systematic review examining the determinants of health workforce emigration from 1970 to 2022 found that most studies originated from low- and middle-income countries such as South Africa and India. The main reasons reported for emigration included the pursuit of better salaries (83.2%), improved professional prospects (81.3%), and enhanced safety conditions (58.9%) [5].

In the case of Peru, the Open Doors Report on International Educational Exchange indicates that the number of Peruvian students choosing to pursue education in the United States increased by more than 10% between 2021 and 2023 [6]. Similarly, data from the Peruvian Ministry of Health show that 12,298 Peruvian physicians emigrated to other countries between 1994 and 2004 [7].

A 2008 study involving students from nine Latin American countries reported that 40.1% intended to practice their profession abroad [8]. In Peru, a 2007 study conducted with 202 final-year medical students from a university in Lima found that 38.3% expressed an intention to emigrate [9]. Another study, conducted in 2010 with 289 recent medical graduates in Lima, reported a prevalence of intention to emigrate of 42.1% [10].

Given this scenario, it is crucial to obtain up-to-date data and to further analyze this issue and its underlying causes in order to inform better policies and strategies that may help mitigate the impact of medical emigration in Peru. Therefore, the objective of the present study was to assess the intention to emigrate after graduation among medical students from two universities in Lima, Peru.

## Methods

### Design and participants

We conducted an analytical cross-sectional study using secondary data. We analyzed the database from a study that aimed to describe the perception of Primary Care Labor (PC) among medical students from two universities located in Lima, Peru. Universidad Peruana Unión (UPeU) and Universidad Peruana Cayetano Heredia (UPCH). This study surveyed third- to seventh-year medical students aged 18 and over. Data collection took place between July and December 2023 at UPeU, and between April and November 2024 at UPCH.

In Peru, the medical education program typically lasts seven years. It consists of three years of theoretical training, followed by three years that combine theoretical coursework with clinical practice, and a final year of medical internship, during which students take on greater responsibilities under the supervision of an experienced physician [11].

### Procedures

The questionnaire used in this study was developed by the authors and was first administered in a pilot test involving 15 students (10 from UPeU and 5 from UPCH) to ensure that all questions were clearly understood before distribution to the target population.

At UPeU, data collection was conducted during academic tutoring sessions in the 2023-II semester. Prior authorization was obtained from the responsible faculty members to carry out the study. A brief explanation of the study’s purpose, relevance, and objectives was presented to the students. The questionnaire was then distributed via a link that included the informed consent form. Participation was entirely voluntary, and informed consent was obtained electronically via a digital form preceding the survey. Each student was given approximately 10 minutes to complete the questionnaire.

At UPCH, due to time constraints between classes or tutoring sessions, a digital data collection strategy was implemented. Permission was obtained through class representatives or academic coordinators, and the study was disseminated via institutional email and official WhatsApp groups for students enrolled during the 2024-I and 2024-II semesters. The message included a detailed description of the study objectives, along with a link to the online survey and the informed consent form. Electronic informed consent was required prior to participation.

The anonymized dataset used for this secondary analysis was accessed on March 3, 2025. The authors did not have access to any personally identifiable information during or after data collection.

### Survey instrument

A self-administered survey was developed and structured into three sections:

1) Sociodemographic Data: This section included variables such as age, sex, academic year, place of birth, current residence, marital status, number of children, previous health-related profession, and university of enrollment.
2) Educational and Career Expectations: This section explored topics such as expected salary, preferred medical specialty after graduation, intention to emigrate after graduation, and experiences related to primary care through coursework, clinical rotations, or close contacts.
3) Perception of PC: To assess students’ perceptions of PC, we used a previously validated 11-item scale covering three key domains. This scale has demonstrated good internal consistency [12].

### Dependent variable

The dependent variable was the self-reported intention to emigrate to another country to practice medicine. This was assessed through a direct question in the survey: “After completing your degree, would you like to emigrate to another country to practice professionally?” Response options were “Yes” or “No.”

### Data analysis

Data were analyzed using STATA version 18.0 (Stata Corporation, College Station, Texas, USA) [13]. Descriptive statistics included absolute and relative frequencies. To assess factors associated with the intention not to emigrate, we performed Poisson regression with robust variance. We estimated crude prevalence ratios (cPR) and adjusted prevalence ratios (aPR), along with their respective 95% confidence intervals (95% CI). Variables with a p-value < 0.20 in the unadjusted analysis were considered for inclusion in the multivariable model, in line with the approach used in exploratory studies [14], and additional variables identified a priori as potential confounders based on previous literature [9,15]. A p-value < 0.05 was considered statistically significant.

### Ethical considerations

The study was reviewed and approved by the Ethics Committee of the Universidad Peruana Unión (UPeU) (Approval Code: 2025-CEUPeU-014). Consent was provided via a digital form preceding the online survey, which participants had to read and affirm before responding. For the present secondary data analysis, the anonymized dataset was accessed on March 03, 2025. The research team had no access to identifiable personal data at any point. All procedures followed the ethical principles outlined in the Declaration of Helsinki.

## Results

A total of 550 surveys were obtained from the database. Thirty-five participants were excluded due to incomplete data and seven due to being in an academic year outside the study scope. Ultimately, 508 participants were included in the analysis. Of these, 314 students were from UPeU (response rate: 77.5% out of 403 enrolled) and 194 from UPCH (response rate: 10.6% out of 981 enrolled).

Among participants, 60.4% were female, 82% were aged between 18 and 25 years, and 53.3% were originally from Lima. Regarding career expectations, 68.7% reported a preference to work in a hospital setting, 50% aimed to pursue a clinical career upon graduation, and 50.6% had a favorable perception of physicians working in PC. 92.1% of students expressed an intention to emigrate abroad to practice medicine after completing their degree, with similar proportions across institutions (91.8% at UPCH and 92.4% at UPeU) (Table 1).

**Table 1:** Characteristics of the medical students included in the study (n = 508).

Characteristic	Total	UPeU - n = 314 n (%)	UPCH - n = 194 n (%)
Sex, female	307 (60.4)	195 (63.5)	112 (36.5)
Age (years)*	23.2 ± 2.6	23.8 ± 2.6	22.1 ± 2.1
18–22 years	201 (39.6)	85 (42.3)	116 (57.7)
23–25 years	221 (43.5)	157 (71.0)	64 (29.0)
26–36 years	86 (16.9)	72 (83.7)	14 (16.3)
Academic year			
3rd–4th year	199 (39.2)	101 (50.8)	98 (49.2)
5th–6th–7th year	309 (60.8)	213 (68.9)	96 (31.1)
Place of birth			
Metropolitan Lima	271 (53.3)	146 (53.9)	125 (46.1)
Province	189 (37.2)	124 (62.6)	65 (34.4)
Foreign country	48 (9.5)	44 (91.7)	4 (8.3)
Birthplace, urban area	489 (96.3)	306 (62.6)	183 (37.4)
Marital status, single	495 (97.4)	302 (61.0)	193 (39.0)
Number of children, none	499 (98.2)	306 (61.3)	193 (38.7)
Has a family member working in PC, no	414 (81.5)	288 (69.6)	126 (30.4)
Has a close friend working in PC, no	239 (47.1)	113 (47.3)	126 (52.7)
Has a teacher working in PC, no	437 (86.0)	309 (70.7)	128 (29.3)
Has taken courses/seminars on PC, no	199 (39.2)	40 (20.1)	159 (79.9)
Has completed an external rotation in PC centers, no	364 (71.7)	250 (68.7)	114 (31.3)
Has done community health promotion volunteer work, no	418 (82.3)	304 (72.7)	114 (27.3)
Second health-related degree, no	495 (97.4)	302 (61.0)	193 (39.0)
Expected monthly salary (in Peruvian soles)			
Less than 7000	201 (39.6)	91 (45.3)	110 (54.7)
Between 7000 and 9000	163 (32.1)	135 (82.8)	28 (17.2)
More than 9000	144 (28.3)	88 (61.1)	56 (38.9)
Expected workplace after graduation			
Hospital	349 (68.7)	251 (71.9)	98 (28.1)
Private clinic	139 (27.4)	59 (42.4)	80 (57.6)
Primary care center	11 (2.2)	4 (36.4)	7 (63.6)
Other	9 (1.8)	0	9 (100.0)
Preferred area of specialization			
Medical	254 (50.0)	156 (61.4)	98 (38.6)
Surgical	237 (46.7)	155 (65.4)	82 (34.6)
Other	17 (3.3)	3 (17.6)	14 (82.4)
Perception of physicians working in PC practice, First tertile	257 (50.6)	202 (78.6)	55 (21.4)
Intention to emigrate abroad for professional practice, yes	468 (92.1)	290 (61.9)	178 (38.1)

* Mean ± standard deviation.

PC: Primary Care.

When analyzing the frequency of intention to emigrate by university and academic year, we observed that this ranged from 78.7% to 100%. At UPCH, the highest percentage was recorded in the sixth year (100%), while at UPeU, the highest frequencies were observed in the fifth and sixth years (96.1% in both cases) (Table 2).

**Table 2:** Intention to emigrate by university and academic year.

Academic year	UPCH Intention to emigrate, n (%)	UPeU: Intention to emigrate, n (%)
3rd	51 (91.1)	37 (78.7)
4th	39 (92.8)	51 (94.4)
5th	62 (89.8)	74 (96.1)
6th	18 (100.0)	75 (96.1)
7th	8 (88.9)	53 (91.4)

In the analysis of factors associated with the intention not to emigrate, the prevalence was lower among students aged 23–25 compared to those aged 18–22 (adjusted prevalence ratio (aPR: 0.35; 95% CI: 0.13–0.91). Conversely, it was higher among students who had completed an external rotation in PC (aPR: 2.10; 95% CI: 1.18–4.10). Additionally, the intention not to emigrate was lower among those who preferred a surgical specialty after graduation (aPR: 0.40; 95% CI: 0.20–0.82) (Table 3).

**Table 3:** Factors associated with the Intention not to emigrate for professional development (n = 508).

Characteristic	Intend to Emigrate (n = 468)	Don’t Intend to Emigrate (n = 40)	Bivariate Analysis		Adjusted Model*	
			PR	95% CI	PR	95% CI
Sex						
Male	186 (92.5)	15 (7.5)	Ref.	–	–	–
Female	282 (91.8)	25 (8.2)	1.09	(0.58 - 2.02)	–	–
Age group						
18–22 years	177 (88.1)	24 (11.9)	Ref.			
23–25 years	215 (97.3)	6 (2.7)	0.22	(0.09 - 0.54)	**0.35**	**(0.13 - 0.91)**
26–36 years	76 (88.4)	10 (11.6)	0.97	(0.49 - 1.94)	1.54	(0.70 - 3.39)
Academic year						
3rd–4th year	178 (89.4)	21 (10.6)	Ref.	–		
5th–6th–7th year	290 (93.9)	19 (6.1)	0.58	(0.32 - 1.05)	1.31	(0.64 - 2.68)
University						
U Peruana Cayetano Heredia	178 (91.7)	16 (8.3)	Ref.	–	–	–
U Peruana Union	290 (92.4)	24 (7.6)	0.92	(0.50 - 1.70)	–	–
Place of birth						
Metropolitan Lima	246 (90.8)	25 (9.2)	Ref	–	Ref	–
Province	175 (92.6)	14 (7.4)	0.80	(0.43 - 1.50)	0.90	(0.48 - 1.68)
Foreign country	47 (97.9)	1 (2.1)	0.22	(0.31 - 1.63)	0.47	(0.08 - 3.34)
Has done community health promotion volunteer work						
No	385 (92.1)	33 (7.9)	Ref.	–	–	–
Yes	83 (92.2)	7 (7.8)	0.98	(0.45 - 2.15)	–	–
Expected monthly salary						
Less than 7000	177 (88.1)	24 (11.9)	Ref.	–	Ref.	–
Between 7000 and 9000	158 (96.9)	5 (3.1)	0.25	(0.10 - 0.65)	0.45	(0.12 - 1.71)
More than 9000	133 (92.4)	11 (7.6)	0.64	(0.32 - 1.26)	0.69	(0.31 - 1.54)
Has a family member working in PC						
No	386 (93.2)	28 (6.7)	Ref.	–	Ref.	–
Yes	82 (87.2)	12 (12.8)	1.88	(0.99 - 3.57)	1.56	(0.61 - 3.98)
Has a close friend working in PC						
No	215 (89.9)	24 (10.1)	Ref.	–	Ref.	–
Yes	253 (94.0)	16 (6.0)	0.59	(0.32 - 1.08)	0.74	(0.30 - 1.83)
Has a teacher working in PC						
No	404 (92.5)	33 (7.5)	Ref.	–	–	–
Yes	64 (90.1)	7 (9.9)	1.3	(0.60 - 2.83)	–	–
Has taken courses/seminars on PC						
No	175 (87.9)	24 (12.1)	Ref.	–	Ref.	–
Yes	293 (94.8)	16 (5.2)	0.43	(0.23 - 0.78)	0.81	(0.38 - 1.71)
Has completed an external rotation in PC centers						
No	344 (94.5)	20 (5.5)	Ref.	–	Ref	–
Yes	124 (86.1)	20 (13.9)	2.53	(1.40 - 4.55)	**2.10**	**(1.08 - 4.10)**
Preferred area of specialization						
Medical	225 (88.6)	29 (11.4)	Ref.	–	Ref.	–
Surgical	227 (95.8)	10 (4.2)	0.37	(0.18 - 0.74)	**0.40**	**(0.20 - 0.82)**
Other	16 (94.1)	1 (5.9)	0.51	(0.07 - 3.56)	0.34	(0.07 - 1.62)
Perception of physicians working in PC labor						
First tertile	235 (91.4)	22 (8.6)	Ref		–	–
Second tertile	80 (93.0)	6 (7.0)	0.81	(0.34 - 1.94)	–	–
Third tertile	153 (92.7)	12 (7.3)	0.84	(0.43 - 1.67)	–	–

PR = Prevalence Ratio; Ref. = Reference category; CI = Confidence Interval, PC = Primary Care.

* Adjusted for academic year, salary expectation, medical area of preference, having a family member or friend related to PC, having taken courses/seminars, having done a rotation in PC, and place of birth.

## Discussion

### Summary of findings

A total of 92.1% of surveyed medical students expressed an intention to emigrate in order to practice their profession abroad (91.8% at UPCH and 92.4% at UPeU). The intention not to emigrate was more frequent among those who had completed an external rotation in primary care and less frequent among students aged 23–25 and those who preferred surgical specialties upon graduation.

### Intention to emigrate

Among students from two universities in Lima, we found that 92.1% responded affirmatively to the question: “After graduating, would you like to emigrate to another country to practice your profession?” This percentage is notably higher than that reported in previous studies conducted in Peru. For instance, a 2007 study of 214 final-year medical students in Lima reported that 38.3% intended to emigrate, based on responses to the question: “Ten years from now, do you see yourself working abroad?” [9]. Similarly, a 2010 study involving 289 recently graduated physicians from a public university in Lima found that 42.2% intended to emigrate, based on the question: “Do you plan to practice medicine outside the country?” [10]. Furthermore, a 2018 study of 341 medical students from a Peruvian university reported an emigration intention rate of 76.5% [16].

The higher percentage found in our study could reflect a growing interest in emigration among undergraduate medical students, driven by factors such as the perception that other countries offer better opportunities for accessing medical residencies or internships at universities and hospitals with stronger training programs and a higher quality of life [17,18]. In addition, digitalization has facilitated access to information about international pre and postgraduate programs, thereby expanding the horizon of possibilities for these professionals. On the other hand, insecurity, low wages, and limited government investment in medical specializations reduce professional development opportunities in Peru [19,20]. This situation contributes to a “brain drain” phenomenon that threatens the sustainability of the national health system [19].

However, the intention to emigrate appears to be lower among healthcare professionals who are already practicing in Peru. A 2014 study using a national public database of 2228 physicians reported an emigration intention rate of 9.7%, based on two questions: “Do you plan to emigrate to another location?” and “To which department or country do you plan to emigrate?” A participant was considered to have emigration intentions if they answered “yes” to the first question and named a country as their planned destination in the second [21]. This suggests that, over time, healthcare professionals may become more integrated into the workforce, strengthen their social and family ties, assume familial responsibilities, and reach a degree of financial stability. These factors may lead to a reevaluation of their priorities and a greater inclination to remain in their home country [22]. Additionally, fear of cultural adaptation and the experience of living alone and far from their usual social environment may also play a role in deterring emigration [23].

Notably, the intention to emigrate was assessed using a single binary question (“After graduating, would you like to emigrate to another country to practice your profession? Yes/No”), which could capture stated aspirations rather than a definite migration. Accordingly, it is likely that not all respondents who answered affirmatively will ultimately emigrate, as the final decision may be influenced by multiple factors, including the complexity of emigration procedures, language proficiency in the destination country, recognition of professional qualifications, availability of employment opportunities in their desired specialty, and the socioeconomic stability of both the country of origin and the receiving country [24,25].

Furthermore, the response rate among students from UPCH was particularly low, which may limit the representativeness of this subgroup. This raises the possibility of selection bias, as students with stronger opinions or a greater interest in emigration may have been more likely to participate. Consequently, the observed estimates could overrepresent emigration intentions within this group, and the findings should be interpreted with caution.

#### Associated factors.

We observed that students who preferred surgical specialties were more likely to express an intention to emigrate. This finding is consistent with a study conducted in five Chinese universities, which included students from second to final year. That study found that those who preferred specialties such as general surgery, plastic surgery, or emergency medicine were more likely to intend to practice abroad, particularly in high-income countries [26]. Similarly, a scoping review published in 2024 reported that students aspiring to pursue surgical specialties tend to have a higher likelihood of emigration compared to those interested in clinical (non-surgical) specialties. This may be partly due to the perception that other countries offer opportunities for surgical training, access to advanced technologies, and greater exposure to specialized procedures. In contrast, clinical specialties may be more firmly established and accessible in the students’ home countries, reducing the perceived need to seek opportunities abroad [27].

### External rotations in primary care

Participation in an external rotation related to Primary Care (PC) was associated with a lower intention to emigrate. This finding aligns with a study conducted among 341 medical students from a Peruvian university, which showed that those with an intention to emigrate abroad were 43% less likely to choose a specialty related to primary care [16]. A 2015 systematic review aimed at identifying factors associated with physician retention in primary care found that curricula and training programs focused on rural practice had a significant impact, with more than 50% of graduates working in rural areas. Furthermore, short-term training programs in underserved areas were shown to foster and retain primary care physicians, even decades after graduation [28]. These findings suggest that direct exposure to primary health care may enhance students’ professional commitment and reduce negative perceptions regarding limited career opportunities and lower income levels in this field [29].

### Limitations and strengths

This study has several limitations that should be considered when interpreting the findings. First, only 194 out of 981 students from UPCH and 314 out of 403 students from UPeU were surveyed. Therefore, generalizations—particularly for UPCH—should be made with caution. Second, all variables were self-reported, which may introduce recall bias. Additionally, data may be subject to response bias, including social desirability bias, potentially influencing how participants reported their intentions and preferences. Third, this was a secondary data analysis based on a dataset that was not originally designed to specifically assess the intention to emigrate among medical students, which limits the depth of the factors evaluated. Finally, due to its cross-sectional design, temporal relationships cannot be established and causal inference is limited; therefore, the observed associations should be interpreted as correlations rather than causal effects.

Despite these limitations, the study offers valuable insights into key aspects such as salary expectations and preferred medical specialties—factors that play a crucial role in students’ professional development and future career paths. Furthermore, it provides an overview of emigration intentions among future physicians, serving as a foundation for further research.

Future studies should explore additional relevant variables, such as socioeconomic status, academic performance, family influence, and the presence of a role model in the healthcare field. They should also investigate students’ preparation and planning for emigration, including awareness of administrative procedures, available programs, and whether their extracurricular training aligns with the requirements of the intended destination country. Additionally, it is essential to analyze the intention to return to the home country and other factors that may shape the decision to emigrate. In this regard, our study highlights the need for continued exploration of the determinants influencing emigration among future healthcare professionals.

## Conclusions

Our study reveals a high prevalence of emigration intention among medical students from the third to the seventh year. We identified associations with being aged 23–25 and with a preference for surgical specialties. However, as intention to emigrate was assessed using a self-reported question, it does not necessarily reflect actual migration behavior, and given the low response rate observed in one of the universities, the findings should be interpreted with caution. This trend contrasts with the lower emigration rates observed among established professionals within the healthcare system, suggesting that the development of social ties and economic stability may reduce the likelihood of emigration. These findings underscore the importance of implementing policies aimed at improving medical training conditions and employment opportunities, with the goal of retaining medical talent and strengthening the national healthcare system.
